# Homeostasis of the Intestinal Mucosa in Healthy Horses—Correlation between the Fecal Microbiome, Secretory Immunoglobulin A and Fecal Egg Count

**DOI:** 10.3390/ani12223094

**Published:** 2022-11-10

**Authors:** Agnieszka Żak-Bochenek, Joanna Bajzert, Dominika Sambor, Natalia Siwińska, Bogumiła Szponar, Łukasz Łaczmański, Paulina Żebrowska, Aleksandra Czajkowska, Maciej Karczewski, Anna Chełmońska-Soyta

**Affiliations:** 1 Department of Immunology, Pathophysiology and Veterinary Preventive Medicine, Faculty of Veterinary Medicine, Wrocław University of Environmental and Life Sciences, C. Norwida 31, 50-375 Wroclaw, Poland; 2 Department of Internal Diseases and Clinic of Diseases of Horses, Dogs and Cats, Faculty of Veterinary Medicine, Wrocław University of Environmental and Life Sciences, C. Norwida 31, 50-375 Wroclaw, Poland; 3 Laboratory of Genomics and Bioinformatics, Hirszfeld Institute of Immunology and Experimental Therapy, Polish Academy of Sciences, 53-114 Wroclaw, Poland; 4 Department of Applied Mathematics, Faculty of Environmental Engineering and Geodesy, Wrocław University of Environmental and Life Sciences, C. Norwida 31, 50-375 Wroclaw, Poland

**Keywords:** secretory immunoglobulin A, short-chain fatty acids, gastrointestinal tract, *Cyathostomum*, mucosal immunity

## Abstract

**Simple Summary:**

The defense effect of the gastrointestinal mucosa in horses, as well as in other mammals, is an extremely complex process and is dependent on many parameters. Mutual communication between the host and the microbiome is essential for intestinal mucosa homeostasis. In horses, in the described system, constant exposure to nematode eggs infection is also important. The presented studies showed the existence of significant relationships between the host response and composition and metabolic activity of microbiome as well as the presence of small parasites in the digestive tract of horses. Greater microbiome diversity correlates with greater production of the neutralizing immunoglobulin A, and a key commensal bacteria phylum in horses, *Firmicutes*, is negatively correlated with fecal egg count.

**Abstract:**

The defensive function of the intestinal mucosa depends both on the ability to secrete immunoglobulin A and communication with the mucus microbiome. In horses, the functioning of this system is also influenced by the presence of nematode eggs. Feces collected from healthy horses were examined to determine the fecal egg count, immunoglobulin A level (ELISA), microbiome composition (Next-Generation Sequencing, NGS, V3–V4 and V7–V9 hypervariable regions *of the 16S rRNA* gene analysis and short-chain fatty acid (SCFA) production ((high-performance liquid chromatography, HPLC). In the taxonomic analysis within the phylum, the following order of dominance was found*: Firmicutes, Bacteroidota, Verrucomicrobiota* and *Fibrobacterota*. The coefficient of phylogenetic diversity of the microbiome positively correlated with both secretory immunoglobulin A (SIgA) [μg/g of feces] (*p* = 0.0354, r = 0.61) and SIgA [μg/mg of fecal protein] (*p* = 0.0382, r = 0.6) and with the number of *Cyathostomum* eggs (*p* = 0.0023, r = 0.79). Important components of the key microbiome in horses, such as phylum *Proteobacteria* and species *Ruminococcus flavefaciens*, were positively correlated with the fecal SIgA (*p* < 0.05). All the obtained results indicate the existence of significant relationships between the host response (SIgA production) and composition and SCFA production in the microbiome as well as the presence of small strongyles in the digestive tract of horses.

## 1. Introduction

The defense effect of the gastrointestinal mucosa in horses, as well as in other mammals, is an extremely complex process and is dependent on many parameters. The physical barrier consists of a mucus layer and closely adjacent mucosal epithelial cells (MECs). The mucous barrier provides the first immune defense barrier for pathogens and anchoring for protective factors [1]. The outer mucous layer is colonized by the intestinal commensal microbiota. The inner layer is responsible for the protection of MECs against bacteria, including commensal ones, via the presence of secretory immunoglobulin A (SIgA) and antibacterial peptides (AMPs) such as defensins or lactoferricins [2]. Their coexistence is known as the “kill zone” [3]. A healthy gastrointestinal microbiota demonstrates a protective effect within the mucosa in three ways: (a) competition for the site of colonization and availability of nutrients; (b) within the kill zone, commensal bacteria stimulate the production of mucins, SIgA and AMPs; (c) by enhancing the host’s immune response to pathogens by stimulating the cells to produce IL-22 (T cells, NK cells) and IL-1β (intestinal monocytes) [4]. The production and quantification of SIgA in the feces may be a marker of gastrointestinal-associated lymphoid tissue (GALT) function. As commensal microflora in the equine hindgut, *Firmicutes, Bacteroidetes* and *Verrucomicrobia* are amongst the predominating phyla [5,6,7,8]. An important function performed by commensal bacteria is the production of short-chain fatty acids (SCFAs). SCFAs are a source of energy, and can also have a protective and repairing effect on intestinal cells. They also promote proliferation and the de novo induction of Treg cells and suppress production of proinflammatory cytokines via macrophages [9]. When discussing the dependencies on the functioning of GALT in horses, one cannot forget about the high exposure of this area to parasites—especially small strongyles, which, despite parasite control programs that include anthelmintic, inhabit the gastrointestinal tract [10]. The interrelationships between the presence of parasites and the functioning of the mucosal “kill zone” in horses have not been fully established.

The main objective of the study was to determine the relationship between the “kill zone” components, such as SIgA and the gut microbiome, as well as the degree of invasion of small strongyles in horses. To address these questions using feces collected from healthy horses, the number of Cyathostomum eggs, SIgA level, microbiota composition and the ability to produce SCFAs were assessed. An additional goal was to try to determine the stool SIgA and SCFAs as potential markers of the functioning of the immune system within the gastrointestinal tract.

## 2. Materials and Methods

### 2.1. Experimental Design

The study was conducted in 60 clinically healthy adult horses of different sex, age (>2.5 y.o.) and breed, housed in three different breeding centers (detailed in Table 1). In stable No1, all test horses were fed equally—they had ad libitum access to grass pasture, high-quality hay, water and mineral licks. In stables No2 and No3, the horses had ad libitum access to a grass pasture, high-quality hay, water and mineral licks; additionally, they were fed concentrated feed according to their needs. In the groups from stables No2 and No3, it was impossible to standardize the way of feeding the horses for the purposes of the study. All horses were routinely dewormed with oral pastes containing ivermectin (18.7 mg/g) and praziquantel (140.3 mg/g) in doses of 1.07 g paste/100 kg body mass. The deworming program was defined for each stable and included deworming twice a year with a two-component preparation process. According to the schedule at the time of study, in stables No1 and No3, the horses were dewormed 6 months before sampling and in stable No2 1.5 months before sampling. According to the information from the owner, none of the horses showed symptoms of gastrointestinal diseases such as colic or diarrhea and had not been treated in the last two months. Feces samples were collected from each horse during spontaneous defecation (immediately after defecation in a volume of 100 mL for further determination and preservation) [11]. The samples were divided and protected by freezing at −80 °C. Immediately after sampling, 4 g of fresh feces was weighted and collected and stored in a refrigerator (2–8 °C) for fecal egg count (FEC) analysis. In stable No1, samples were additionally collected from the center of the stool clump in a sterile manner to avoid contamination with environmental factors: 1 g of feces was weighed and suspended in 5 mL of 70% ethanol and a sample of about 1 g of feces was placed in sterile Eppendorf tubes [12]. Both types of samples were frozen (−20 °C) immediately after sampling and then in −80 °C after transport. Additionally, in connection with the routine blood tests in stable No1, blood samples were collected from the external jugular vein into a tube with a clot activator and then centrifuged (10 min, 1500 g) to obtain serum and frozen (−80 °C). The following analyses were carried out in fecal samples from horses from the No1 stable, due to the uniform method of maintenance and feeding: FEC, fecal protein, fecal SIgA, fecal microbiome and SCFA production. In the remaining samples, due to the difficulty of systematizing conditions, the fecal microbiome was not determined.

### 2.2. Methods

#### 2.2.1. Fecal Egg Count (FEC)

Modified McMaster method: Fecal egg count was performed to establish the number of small strongyle (*Cyathostomum* spp.) eggs in equine feces. FEC was performed with a modified McMaster method, as has been described before [13]. This method was chosen because it is one of the most practical ways to estimate the level of infection. Briefly, the fecal examination was performed, followed by centrifugation—an enhanced method using a sugar–salt flotation solution with a specific gravity of 1.3 g/mL [13]. The detection limit of the method was 20 eggs per gram (EPG) [13]. For the value of 0 eggs in the field of view, a value of 20 was adopted, to minimize statistical mistakes.

#### 2.2.2. Fecal Protein Analysis

After thawing at room temperature, the fecal samples were prepared as described in the literature according to Marr et al., using proprietary modifications [14]. From wet feces samples, 1 g (±0.05 g) of each was weighed and suspended in 5 mL of phosphate-buffered saline (PBS) solution (pH~7.4). In order to mix them thoroughly and release the proteins, the samples were subjected to the following procedure: shaking (3 min) and resting (15 min), twice. Samples were then centrifuged for 20 min at 1600× *g*. The resulting centrifugation supernatant was collected, filtered through a syringe filter (syringe filter, 0.22 µm, ∅33 mm, sterile, TPP, 99722) and then placed in an Eppendorf tube containing a protease inhibitor cocktail (10 µL/1000 µL supernatant; Calbiochem Protease Inhibitor Cocktail Set I 539131). Samples were centrifuged again for 15 min at 3260× *g*. A total of 600 µL of supernatant was withdrawn for protein content assessment, and the excess was frozen at −80 °C for further SIgA analysis. Analysis of fecal protein of the prepared samples was performed using the commercial Pierce BCA Protein Assay Kit (ThermoFisher Scientific, Rockford, IL, USA) according to the manufacturer’s instructions. After the initial determinations were made, the optimal dilution of the samples was found at the level of 1:25.

#### 2.2.3. Serum IgA and Fecal SIgA Analysis

The assay was performed in the supernatant obtained as described in point 2.2 and serum samples using a commercially available ELISA kit (IgA Horse ELISA Kit, Abcam Cat. No. ab190530, Cambridge, UK) according to the manufacturer’s instructions. The fecal supernatant samples and serum samples were thawed at room temperature and then diluted to 1:5 and 1:10,000, respectively. The intra-assay CV reported in these studies was CV = 1.69%, the inter-assay CV = 2.35%.

#### 2.2.4. Fecal Short-Chain Fatty Acid (SCFA) Analysis

Analysis of derivatized stool extracts by high-performance liquid chromatography (HPLC) was performed as previously described [15]. Briefly, freshly picked stool samples were extracted with 70% ethanol. Debris was removed by centrifugation and 500 μL of supernatant was transferred to a new tube and mixed with 50 μL of internal standard (2-ethylbutyric acid, 200 mM in 50% aqueous methanol), 300 μL of dehydrated pyridine 3% *v*/*v* (Merck, St.Luis, MO, USA) in ethanol, 300 μL of 250 mM N-(3-dimethlaminopropyl)-N′;-ethylcarbodiimide hydrochloride (Sigma-Aldrich, St.Luis, MO, USA) in ethanol and 300 μL of 20 mM 2-nitrophenylhydrazine hydrochloride (Sigma-Aldrich, St. Luis, MO, USA) in ethanol. Samples were incubated at 60 °C for 20 min and then mixed with 200 µL of potassium hydroxide solution (15% *w*/*v* with water, and used at a potassium hydroxide solution/methanol ratio of 80/20) as a reaction stopper and reacted at 60 °C for 20 min. After cooling, the mixture was shaken with 3.0 mL of phosphoric acid aqueous solution (0.5 mol/L) and 4.0 mL of ether two times for three minutes for extraction. The organic phase was extracted by shaking with 4 mL of diethyl ether and transferred to a new glass conical containing water to extract any remaining aqueous compounds. The obtained fatty acids hydrazide was dissolved in 100 µL of methanol and 20 µL was subjected to HPLC [15]. HPLC was performed using 1525 Binary HPLC Pump with a 2489 UV/Visible (UV/Vis) Detector and Phenomenex Gemini 5 µm C18 110 A (150 × 4.6 mm). The mobile phase was composed of acetonitrile–methanol–water (30:16:54), and the pH was adjusted to 4.5 with 0.1% trifluoroacetic acid. The column temperature was 50 °C; flow rate, 1.0 mL/min; and measurement wavelength, 400 nm [15]. The following SCFAs were determined: lactic acid, acetic acid (C2), propionic acid (C3), butyric acid (C4), isobutyric acid (iC4), valeric acid (C5) and isovaleric acid (iC5). The results are presented in the unit [mmol / 1g of feces]. As an important determinant of fibrolytic activity, the volatile fatty acid (VFA) ratio was counted, according to the formula: (C2 + C4)/C3 [16].

#### 2.2.5. Microbiome Analysis

Microbial DNA was extracted using QIAamp PowerFecal Pro DNA Kit (Qiagen, Hilden, Germany) from frozen stool material from 12 horses. *16S rRNA* gene amplicon libraries were prepared according to the standard protocol using QIAseq 16S/ITS Region Panels (Qiagen, Hilden, Germany) for V3–V4 and V7–V9 regions. Paired-end sequencing of the amplicons was performed on a MiSeq instrument, using MiSeq Reagent Kit v3, 600 cycles (Illumina, San Diego, CA, USA). To perform bioinformatic analysis of the microbiomes, QIIME2 2021.8 with the supplementary plugins was used [17]. A custom script that uses cutadapt was used to cut V3–V4 and V7–V9 primers and execute demultiplexing [18]. Using the summary method from the demux plugin, the evaluation of the quality of the reads was performed. The dada2 plugin for paired-end reads was used for fortrimming, denoising, dereplication and chimera filtering [19]. The construction of a phylogenetic tree was performed using the q2-phylogeny align-to-tree-mafft-fasttree plug procedure [20,21]. The procedure internally uses the mafft method to align multiple sequences and then masks the highly variable positions, and subsequently fasttree is used to construct a phylogenetic tree. For the V3–V4 region, rarefaction (subsampling without replacement) was conducted up to 22.039 sequences per sample and up to 18.799 for the V7–V9 region. To estimate the diversity, alpha (α) (Shannon’s diversity index, observed features, Faith’s phylogenetic diversity, Pielou’s evenness) and beta (β) (Jaccard similarity index, Bray–Curtis dissimilarity, unweighted UniFrac, weighted UniFrac), as well as to perform principal coordinate analysis (PCoA), the q2-diversity plugin was used [21,22,23,24,25]. The naive Bayesian classifiers trained on fragments V3–V4 and V7–V9 of the *16S rRNA* gene sequences derived from the SILVA 138 SSURef NR99, and the fit-classifier-naive-bayes method from the feature-classifier plugin was used to assign taxonomy to ASVs (amplicon sequence variants) [26,27,28]. The demultiplexed fastq data are publicly available on the SRA database under the accession number SAMN31431713 [26,27,28].

#### 2.2.6. Statistical Analysis

Descriptive statistics are presented as mean and standard deviation. Analysis of the differences in parameters between stables was performed using the Kruskal–Wallis test with post hoc analysis using Holm correction. Analysis of correlation between parameters both within and between groups of parameters was performed using Spearman correlation coefficient. Spearman’s rank correlation coefficient was performed to correlate all measurements with α-diversity metrics, and the Mantel test with 999 permutations in the case of β-diversity. False-discovery rate correction was applied to control type I error. Calculations were made using the R package for Windows (version 4.2), and for bioinformatic analysis of the microbiomes, QIIME2 2021.8 with the supplementary plugins was used [17]. Statistical results were considered significant when the *p*-value was below 0.05.

## 3. Results

### 3.1. Fecal Egg Count, Serum IgA and sIgA Analysis Results

The results of the tests for fecal egg count, serum IgA, SigA and fecal protein content are presented in Table 2.

In stable No2, significantly lower concentrations of SIgA [μg/g of feces] and SIgA [μg/mg of fecal protein] were observed in comparison to stable No1 (*p* = 0.043, *p* = 0.013, respectively) and No3 (*p* = 0.043, *p* = 0.046, respectively). There was no correlation between the parameters (FEC, SIgA, fecal protein) and the age of the horses.

### 3.2. SCFA’s Analysis Results

The mean values of SCFAs and % contents in the feces of horses from stable No1 are presented in Table 3.

C2, C3 and C5 concentration showed a dependence on body weight (*p* = 0.021, r = −0.65 and *p* = 0.049, r = −0.58 and *p* = 0.035, r = −0.61, respectively). The VFA ratio correlated with age (*p* = 0.043, r = 0.59). The VFA ratio was dependent on C2 level (*p* = 0.00144, r = 0.83). There were no correlations between SCFAs and FEC, the level of SIgA in the feces, and IgA in the serum.

### 3.3. Microbiome Analysis Results

#### 3.3.1. V3–V4 Hypervariable Regions of the 16S rRNA Gene Analysis

**Figure 1:**
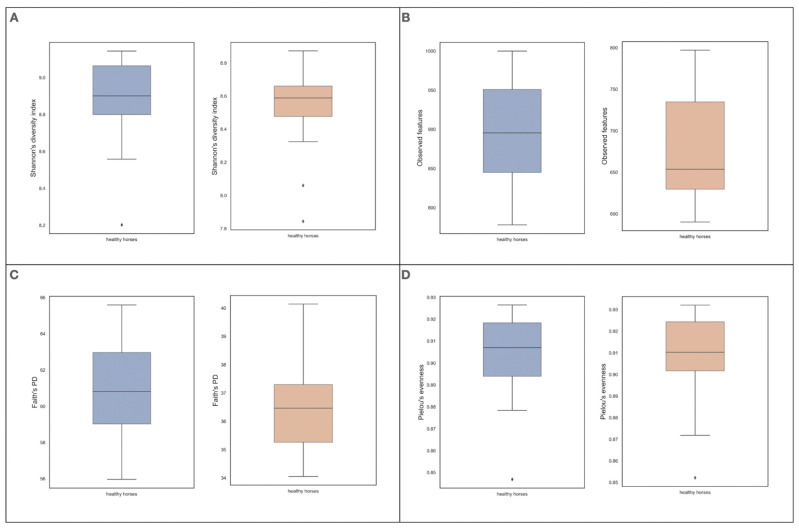
Alpha-diversity measures based on hypervariable regions of *16S rRNA* gene, blue boxplot on the left side of each panel based on V3–V4 and orange boxplot on the right side based on V7–V9 hypervariable region, respectively. (**A**) Shannon’s diversity index: V3–V4 8.849 (0.276), 8.901 (0.265) and V7–V9 8.510 (0.299), 8.588 (0.183); (**B**) observed features: V3–V4 898 (71.334), 895.5 (106) and V7–V9 674.167 (68.911), 653.5 (105.25); (**C**) Faith’s phylogenetic diversity: V3–V4 60.915 (2.760), 60.809 (3.939) and V7–V9 36.53 (1.849), 36.462 (2.042); (**D**) Pielou’s evenness: V3–V4 0.902 (0.023), 0.907 (0.024) and V7–V9 0.906 (0.024), 0.910 (0.023). Legend: mean (SD), median (IQR).

The α- diversity parameters (Pielou’s evenness, Shannon’s diversity index, Faith’s phylogenetic diversity and observed features) for the V3-V4 region are presented in Figure 1. Faith’s PD correlated with FEC (*p* = 0.0023, r = 0.79), SIgA [μg/g of feces] (*p* = 0.0354, r = 0.61), SIgA [μg/mg of fecal protein] (*p* = 0.0382, r = 0.6) and serum IgA [μg/mL] (*p* = 0.059, r = 0.54, trend). Correlations of α-diversity parameters and β-diversity with SIgA are presented in Table 4. Jaccard similarity correlated with iC4 (*p* = 0.048, r = 0.31) and C4 (*p* = 0.026, r = 0.36), weighted UniFrac correlated with C2 (*p* = 0.007, r = 0.48), and unweighted UniFrac correlated with C2 (*p* = 0.021, r = 0.43), C3 (*p* = 0.02, r = 0.4), C4 (*p* = 0.02, r = 0.41) and C5 (0.018, 0.38).

**Figure 2:**
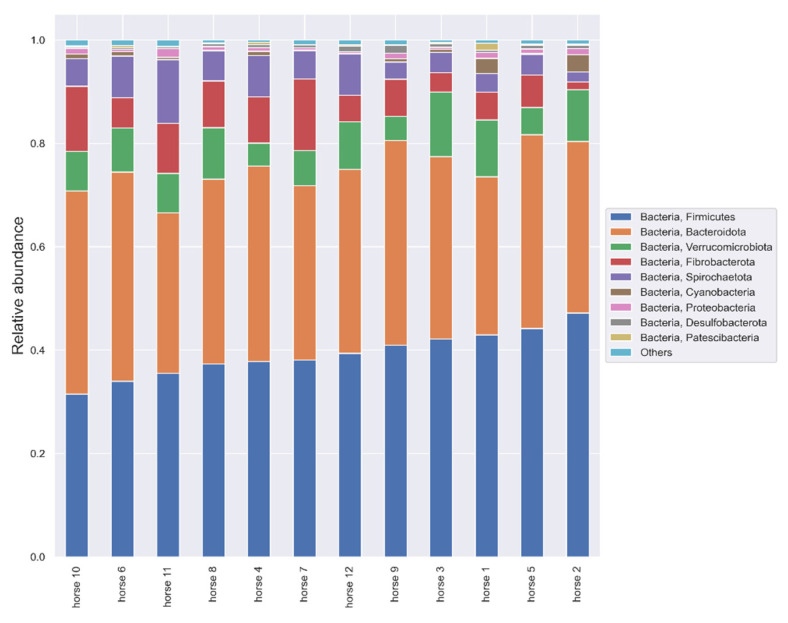
Relative abundance of microbiota from horse feces at the phylum level. Taxonomy based on V3–V4 hypervariable region of 16S rRNA gene. Phyla whose relative abundance accounts for less than 0.01 are found in Others.

In the taxonomic analysis within the phylum, the following order of dominance was found: *Firmicutes* (38.627%, sd: 44.08), *Bacteroidota* (36.383%, sd: 3.453), *Verrucomicrobiota* (8.306% sd: 2.640) and *Fibrobacterota* (7.463% sd: 3.584). The remaining phyla and their relative abundance are shown in Figure 2.

**Figure 3:**
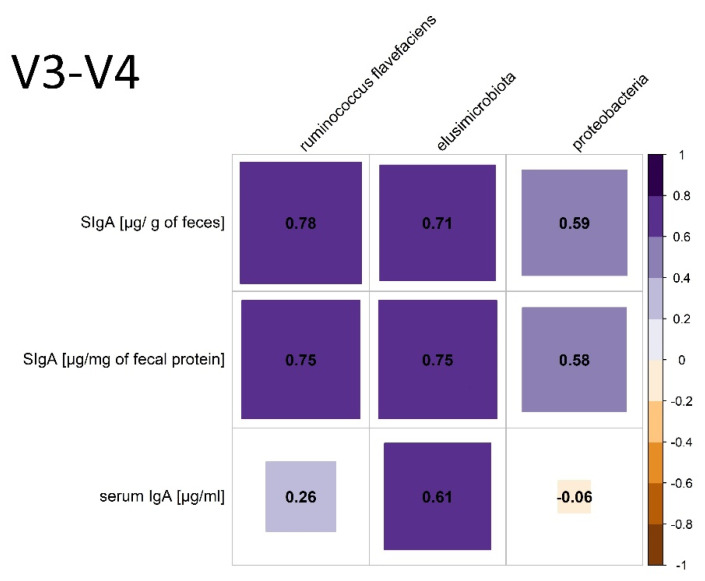
Relationships between important phyla and species and the level of SIgA in the feces and IgA in the serum. *p* < 0.05.

The following positive correlation between individual phyla and species and the production of SCFAs was found: C2 and *Firmicutes* (*p*= 0.0259, r = 0.65). Negative correlations were noticed between C2 and *Bacteroidota* (*p* = 0.022, r = −0.66); iC4 and *Bacteroidota* (*p* = 0.0348, r = −0.62); C4 and *Spirochaetota* (*p* = 0.0484, r = −0.59); and C4 and *Escherichia Shigella p* = 0.053, r = −0.57 (trend). The VFA ratio correlated with *Firmicutes* (*p* = 0.0091, r = 0.73) and *Fibrobacterota* (*p* = 0.0041, r = −0.78). The relative frequency of the genus *Ruminococcus flavefaciens* correlated negatively with C4 (*p* = 0.032, r = −0.63). The relative frequency of genus *Clostridium* s. *stricto* correlated with body weight (*p* = 0.0096, r = 0.71). There were negative correlations between FEC and the relative frequency of *Firmicutes* (*p* = 0.003, r = −0.78) and *Desulfobacterota* (*p* = 0.0443, r = −0.59). Relationships between some phyla and species and the level of SIgA in the feces and IgA in the serum were found; they are presented in Figure 3.

#### 3.3.2. V7-V9 Hypervariable Regions of the 16S rRNA Gene Analysis

The VFA ratio positively correlated with Shannon entropy (*p* = 0.019, r = 0.68). The α-diversity parameters (Pielou evenness, Shannon entropy, Faith’s PD and the number of observed features) for the V7–V9 region are presented in Figure 1. Correlations for α-diversity parameters and β-diversity are presented in Table 4. C2 correlated positively with Bray–Curtis dissimilarity *p* = 0.048, r = 0.3, the Jaccard similarity index *p* = 0.045, r = 0.34, weighted UniFrac *p* = 0.017, r = 0.44 and unweighted UniFrac *p* = 0.02, r = 0.35. C4 correlated with the Jaccard similarity index *p* = 0.04, r = 0.32.

**Figure 4:**
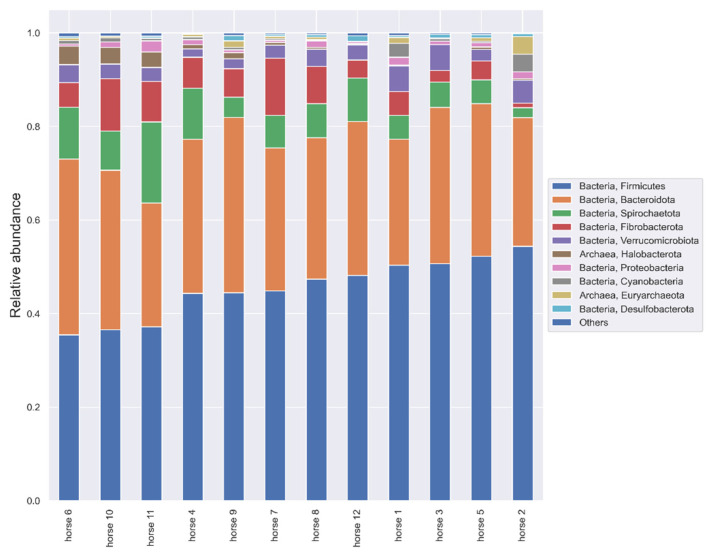
Relative abundance of microbiota from horse feces at the phylum level. Taxonomy based on V7–V9 hypervariable region of 16S rRNA gene. Phyla whose relative abundance accounts for less than 0.01 are found in Others.

In the taxonomic analysis within the phylum, the following order of dominance was found: *Firmicutes* (43.819%, sd: 6.382), *Bacteroidota* (33.696%, sd: 3.751), *Verrucomicrobiota* (7.534% sd: 3.883) and *Fibrobacterota* (6.404% sd: 3.444). The remaining phyla and their relative frequency are shown in Figure 4.

**Figure 5:**
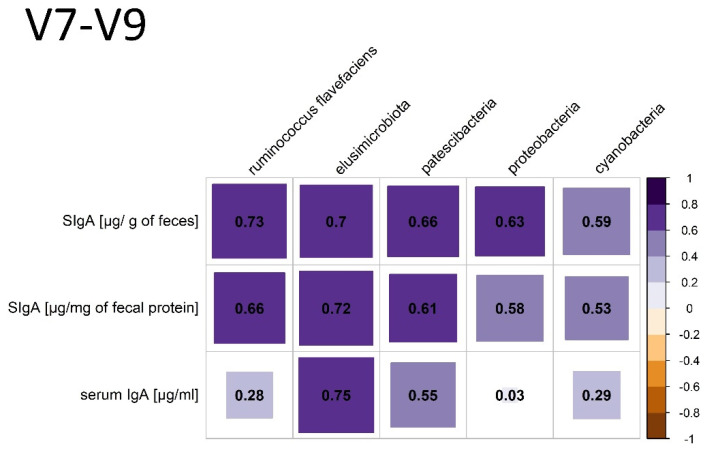
Relationships between important phyla and species and the level of SIgA in the feces and IgA in the serum. *p* < 0.05.

Compared to the analysis of the V3–V4 region, the phylum *Armatimonadota* was observed and no *Chlorflexi* appeared. The following positive correlations between individual phyla, species and the production of SCFAs were found: C2 and *Firmicutes* (*p*= 0.0488, r = 0.59); C5 and *Actinobacteriota* (*p* = 0.0091, r = 0.73); iC5 and WPS_2 (*p* = 0.0107, r = 0.7). A negative correlation was noticed between C2 and *Fibrobacterota* (*p* = 0.0458, r = −0.59); C4 and *Halobacterota* (*p* = 0.0324, r = −0.63); iC5 and *Halobacterota* (*p* = 0.0259, r = −0.65); lactic acid and *Ruminococcus flavefaciens* (*p* = 0.013, r = −0.71). There were negative correlations between FEC and the relative frequency of *Firmicutes* (*p* = 0.0389, r = −0.60) and a positive correlation between FEC and *Elusimicrobiota* (*p* = 0.0295, r = 0.63). Relationships between some phyla and species and the level of SIgA in the feces and IgA in the serum were found; they are presented in Figure 5.

## 4. Discussion

In the presented study, the relationships between the production of SIgA, the microbiome and the degree of *Cyathostomum* infection in the gastrointestinal tract of healthy horses were assessed. The mean content of SIgA in the feces of healthy horses was established at the level of 0.23 (±0.23) μg/mg of fecal protein. Equine researchers have focused only on the potential use of SIgA in the feces as a stress marker; however, the available results do not clearly confirm this relationship [14]. Studies in wild equidae in Africa have considered fecal SIgA as a parasitic nematode-associated antibody, which suggests that equids may develop an IgA response to nematode egg production when the host is in good condition [29]. Studies on the measurement of fecal immunoglobulins and their dependence on GI pathology have been carried out in dogs, and they have shown a decrease in fecal SIgA in the course of IBD [11]. The presented studies did not find any correlation between FEC and the amount of SIgA in the feces of healthy horses. However, we did not assess the presence of specific antibodies against larval or adult *Cyathostomum*. Significantly lower values of SIgA concentration in the feces from stable No.2 in relation to stables No.1 and No.3 (taking into account the time that had passed since the last deworming) seem to suggest a correlation between the absence of parasites in the gastrointestinal tract and a decrease in SIgA production, but confirmation of this requires further research.

The study of the microbiome was carried out on 12 horses housed in one breeding facility. This made it possible to completely unify the nutritional and climatic conditions; these horses had constant, 24 h access to a pasture and high-quality hay, water and mineral licks. They received no concentrated feed. The dominance of individual phyla was similar to that presented in the literature, usually in the following order: *Firmicutes* (>60%), *Bacteroidetes*, *Proteobacteria* and *Verrucomicrobia* [5,30,31]. However, in the presented work, the phylum *Firmicutes* was only about 35% in equilibrium with *Bacteroidetes*, and *Fibrobacterota* appeared as the third phylum. These differences may be due to the type of diet, which can significantly affect the composition of the microbiome [6].

In assessing the gut microbiome in specific species, it is important to know the core microbiota, which should be consistent throughout a stable in terms of bacterial components, including key microorganisms and their functions [8]. The core community at the operational taxonomic unit (OTU) level in feces is defined as “being present in all samples included in the study at least 0.1% relative abundance” [32]. According to the definition of core microbiota in our study, the phyla *Firmicutes*, *Bacteroidota*, *Verrucomicrobiota*, *Fibrobacterota*, *Spirotracheota*, *Proteobacteria*, *Cyanobacteria* and *Desulfobacteria*, and at the family level, *Lachnospiraceae* and *Ruminococcaceae*, constitute the core. However, due to high heterogeneity of the equine core microbiome, the “key microbiome” should be distinguished—species crucial for the proper functioning of the digestive tract [6]. In horses, the three most important phyla are *Firmicutes*, *Proteobacteria* and *Verrucomicrobia* due to having the greatest relevance in digestion, rather than due to their quantitative dominance [6]. Within the phylum *Firmicutes*, the *Lachnospiraceae*, being responsible for the production of butyrate, as well as the *Ruminococceaceae* and *Fibrobacteraceae*, with cellulolytic properties, are the most important for the proper functioning of the digestive tract of horses [6]. In the present study, the *Lachnospiraceae* correlates negatively with *Escherichia Shigella* species (*p* = 0.0041, r = −0.78), which may suggest the protective activity of this group. *Ruminococcus flavefaciens*, a representative of this phylum, shows a strong positive correlation with the amount of SIgA in the feces, which suggests their significant role in the regulation of SIgA production. *Ruminococcus flavefaciens* was the predominant cellulolytic bacterial species in the pony and donkey cecum [33]. The authors are aware that the evaluation of correlations at the species level with amplicon sequencing is rarely reliable; however, among the phyla, *Ruminococcaceae* was the dominant strain, also being relevant to the physiology of the horse’s digestive tract. The second important phylum, whose role is not fully understood, is *Proteobacteria*, which is dominant in the equine small intestine. In the feces of the examined horses, it constituted 0.8–0.97% relative abundance [6]. Additionally, the relative prevalence of *Proteobacteria* moderately positively correlates with the amount of SIgA in the stool. *Proteobacteria*, as a group of Gram-negative bacteria often carrying LPS, is one of the possible fecal markers of intestinal homeostasis [16]. Many studies show that *Proteobacteria* species constitute the largest fraction of SIgA-coated microorganisms [34,35]. Additionally, intestinal *Proteobacteria* may influence the abundance of IgA-producing plasma cells in bone marrow and IgA in serum [35]. In the present study, however, no correlation was found between fecal *Proteobacteria* and serum IgA. Studies in mice have also shown that IgA specific for γ-*proteobacteria* can regulate the maturation of the intestinal microflora [36]. In this species, the *Proteobacteria* cluster will decrease with age, but in horses, the proportion of *Proteobacteria* (*Enterobacteriaceae*) increases 50 days after birth and remains unchanged in adults [7,36]. The third “key phylum” described in horses is *Verrucomicrobia*, which primarily colonizes the hindgut. The genus *Akkermansia* is described as being among those most important for the functioning of the mucosa. Its mucin-degrading action helps to maintain the integrity of the mucin layer and decrease inflammation [6]. *Akkermansia mucinophila*, a member of the phylum *Verrucomicrobia*, is highly enriched in the IgA+ fraction in humans [33]. However, in the presented study, there were a lack of correlations between *Akkermansia* species and SIgA in equine feces. In the V7–V9 region analysis, there was also a correlation between SIgA and *Cyanobacteria (Gastranaerophilales)*, whose exact role in animal gut is unknown, but they are thought to have a beneficial effect for their hosts as a source of vitamins B and K [37]. However, there are no data in the literature on the significance of the other phyla, i.e., *Patescibacteria* and *Elusimicrobia*, which are also correlated with SIgA.

Diversity parameters can be used to accurately assess and analyze the variation in and composition of the gut microbiota. In the case of the presented results, the α-diversity was assessed, which made it possible to measure microbiome diversity within a single sample. To assess various aspects of community heterogeneity, the following were used: Shannon’s diversity index (indirect richness), observed features (direct richness), Faith’s phylogenetic diversity (diversity) and Pielou’s evenness (uniformity). By analyzing V3–V4 hypervariable regions of the *16S rRNA* gene, a correlation was demonstrated between indicators of evenness and richness and GALT markers. Faith’s phylogenetic diversity, which takes into account the sum of the branch lengths of a phylogenetic tree connecting all species (so a higher number means more branches, more richness and a more diverse community), positively correlated with both the number of *Cyathostomum* eggs and SIgA. This indicates a strong relationship between the microbiome and SIgA in the “kill zone” and the microbiome and the presence of parasites in the digestive tract of horses. Positive correlations also occurred between the amount of SIgA in the feces and the individual β-diversity metrics. β-diversity quantifies dissimilarities between communities (samples) and allowed us to determine whether the selected factors were related to the microbiome, and the obtained results suggest a clear relationship between the microbiome and GALT activity in healthy horses.

In terms of taxonomy analysis, the performance of *16S rRNA* hypervariable regions varies [38]. In this study, we separately analyzed two regions of the *16S rRNA* gene to estimate microbial community diversity. The resolution of these two fragments differed. On the basis of the hypervariable V3–V4 fragment, 4759 features were identified, and for the V7–V9 fragment, 3921.

The production of SCFAs by bacteria in the digestive tract of horses is closely related to their use as an energy source [30]. It is worth emphasizing that in humans, only about 5% of SCFAs produced in the large intestine is excreted in the feces, and the remaining 95% is absorbed by intestinal epithelial cells [39]. However, determination of their content in stools can provide useful information on the functioning of the microbiome. SCFAs are a kind of communicator between the microbiome and the immune system and are responsible for maintaining the balance in the anti- and pro-inflammatory reaction, including by signaling through the complex of free fatty acid receptors (GPRs) [9]. A special property of SCFAs is the induction of T-regulatory cells (Treg), which takes place through the inhibition of the enzyme histone deacetylase. The greatest inhibitory potential is seen for butyric acid, which causes proliferation and increases the functional capabilities of Treg cells [9]. Acetate is also a source of energy for regenerative and repair processes in tissues, and propionate is a precursor of gluconeogenesis. Analyzed SCFAs showed no correlation with SIgA in the feces of the tested horses. In the presented studies, the share of butyrate among all SCFAs was above 30%, which does not coincide with the results presented in the literature, where its average share does not exceed 6% [16,40]. A hay-only diet and access to pasture could be considered potential causes of these differences, but this issue requires further research. However, the VFA ratio, which is a relevant marker of fibrolytic activity [16], was about 3.473 in the presented study, which is near the normal value in the hindgut of horses fed a high-fiber diet (4.3 to 6.0) [16]. The VFA ratio correlates positively with C5 (*p* = 0.04, r = 0.61), which is in opposition to the results of Raspa et al., where a low level of valeric acid was associated with a fiber-rich diet [41]. However, in the presented study, the C5 value was very low in all horses. Based on data from Nadeau et al., valerate may show a pro-inflammatory effect and could be involved in gastric ulcer pathogenesis in horses; however, this cannot be compared to the current state in the gut [42]. *Bacteroidota* negatively correlated with iC4, which may suggest their pro-inflammatory action in horses, which was also mentioned in the work of Mach et al. They were further described to increase in number in the case of inflammatory bowel disease in humans [43,44].

It seems to be very difficult to try to answer the question of how the moderate presence of parasites influences the maintenance of homeostasis and the proper functioning of the mammalian gastrointestinal tract. There was no correlation between the degree of worming and SIgA. Studies show an increase in the α-diversity of the microbiome in the case of moderate growth parasites in both humans, non-human primates and horses (trend) [45,46,47]. In studies conducted by Peachey et al., the presence of parasites did not affect the relative frequency of phyla and the species representing the core of the microbiome [47]. A study by Walshe et al. covering the effects of anthelmintic treatment on the composition of the intestinal microbiota showed a decrease in the alpha and beta diversity of the fecal microbiota on day 7 after treatment, which reversed by day 14. These changes were accompanied by an increase in inflammatory biomarkers [48]. The use of anthelmintic drugs increases the relative abundance of *Deferribacter* spp. and *Spirochaetes* spp. and causes disturbances in carbohydrate and fiber metabolism [49]. Studies by Mach et al. indicated that *Clostridiales* were significantly associated with parasite egg counts, whereas *Bacteroides*, which is considered pro-inflammatory, was negatively associated with parasite egg counts [44]. Subsequent studies by Clark et al., 2018 showed a reduction in the phyla *Ruminococcus*, *Clostridium* and *Lachnospiraceae* during the egg burden in horses susceptible to infection [50]. The current research also showed a strong positive correlation between Faith’s diversity and the number of strongyle eggs, and at the same time indicates a negative dependence of FEC and the frequency of *Firmicutes* and a negative trend between FEC and *Lachnospiracea* (*p* = 0.0712). In summary, it can be concluded that horses may have correlations between the degree of shedding (also known as the sensitivity to *Cyathostomum* infections) and the composition of the microflora and GI homeostasis. Additionally, the use of anthelmintic drugs affects the fecal microbiome. Considering the latest indications regarding deworming among only “high shedders” horses, the preliminary conclusions are in line with this approach [10].

The limitations of the presented paper certainly include the small number of individuals. In order to have a broader understanding of the issue, it would be appropriate to conduct a larger study, taking into account the different maintenance routines and diets in selected groups.

## 5. Conclusions

In conclusion, all the obtained results indicate the existence of significant relationships between the host response (SIgA production) and the presence of small strongyles in the digestive tract of horses. The most important factor seems to be the correlation between Faith’s PD or “Key microbes” and SIgA in feces. It seems clear that the correct composition and relationship within the “kill zone” is the most important protective barrier, and its breakage may result in pathologies. In the foreseeable future, the authors plan to extend the analysis to individual parts of the gastrointestinal tract.

## Figures and Tables

**Table 1:** Detailed description of stables, horses and analysis.

Stable No	Number of Horses	Mean Age	Last Deworming	Analysis
1	12	Mean 15.5 (sd = 6.6)	6 months	Serum IgA, FEC, Fecal protein, Fecal SIgA, SCFAs, fecal microbiota
2	6	Mean 12.3 (sd = 4.4)	1.5 months	FEC, Fecal protein, Fecal SIgA
3	42	Mean 10.9 (sd = 7.2)	6 months	

Legend: IgA—immunoglobulin A; FEC—fecal egg count; SIgA—secretory immunoglobulin A; SCFAs—short-chain fatty acids.

**Table 2:** The results of the tests for fecal egg count (FEC), serum IgA, SIgA and fecal protein content.

Stable No.	n	Body Weight [kg]	FEC [EPG] ^1^	Serum IgA [µg/mL] ^1^	Protein [mg/g of Feces] ^1^	SIgA [μg/g of Feces] ^1^	SIgA [μg/mg of Fecal Protein] ^1^
1	12	**610.9 (107.5)**	**80.0 (96.1)**, 20.0 (20.0, 140.0)	**973.9 (532)**, 949.3 (646.7, 1267.4)	**15.4 (1.8)**, 15.8 (15.2, 16.5)	**4.2 (3.1)**, 3.2 (1.8, 6.0)2	**0.269 (0.181)**, 0.24 (0.11, 0.36)
2	6	N/A	**20.0 (0.0)**, 20.0 (20.0, 20.0)	N/A	**18.0 (2.8)**, 17.2 (16.0, 19.0)	**1.2 (1.2)**, 8.8 (0.4, 1.2)	**0.06 (0.06)**, 0.04 (0.02, 0.07)
3	42	N/A	**375.7** **(466.7)**, 190 (20.0, 650.0)	N/A	**20.0 (5.0)**, 19.3 (15.6, 23.7)	**4.45 (4.62)**, 2.91 (1.36, 6.09)	**0.25 (0.26)**, 0.14 (0.08, 0.31)
Total	60	N/A	**281.0 (417.9)**, 60 (20.0, 365.0)	N/A	**18.9 (4.7)**, 17.9 (15.4, 22.3)	**4.1 (4.2)**, 2.9 (1.3, 5.1)	**0.23 (0.23)**, 0.14 (0.07, 0.30)

Legend: IgA—immunoglobulin A; FEC—fecal egg count; SIgA—secretory immunoglobulin A; EPG—eggs per gram; N/A—not applicable; ^1^ **mean (SD)**, median (IQR).

**Table 3:** The results of short-chain fatty acid analysis.

SCFAs [mmol/1 g of Feces]	Lactic Acid	C2	C3	iC4	C4	iC5	C5	VFA Ratio
**Mean (SD)**, Median (IQR)	**3.332 (1.730)**, 3.437 (2.144, 4.097)	**17.365 (7.030)**, 14.735 (13.029, 18.567)	**9.606 (3.984)**, 8.408 (7.121, 10.501)	**3.726 (3.914)**, 2.327 (1.724, 3.819)	**16.932 (12.737)**, 11.002 (8.980, 20.601)	**1.302 (2.208)**, 0.000 (0.000, 2.242)	**2.330 (1.603)**, 1.824 (1.209, 2.747)	**3.473 (0.433)**, 3.446 (3.141, 3.625)
% SCFAs (mean)		33.88	18.74	7.27	33.03	2.54	4.54	

Legend: SCFAs—short-chain fatty acids; C2—acetic acid; C3—propionic acid; iC4—isobutyric acid; C4—butyric acid; iC5—isovaleric acid; C5—valeric acid; VFA ratio—volatile fatty acid ratio. mean (SD) is bolded.

**Table 4:** Correlations between β-diversity parameters and SigA in feces for analysis of V3–V4 and V7–V9 hypervariable regions of the *16S rRNA* gene. X—without significant.

	β-Diversity Parameters	SIgA [μg/g of Feces]	SIgA [μg/mg of Fecal Protein]
V3–V4	Bray–Curtis dissimilarity	*p* = 0.002, r = 0.59	*p* = 0.004, r= 0.49
	Jaccard similarity index	*p* = 0.002, r = 0.55	*p* = 0.004, r = 0.44
	Weighted UniFrac	*p* = 0.002, r = 0.33	x
	Unweighted UniFrac	*p* = 0.007, r = 0.41	*p* = 0.048, r = 0.29
V7–V9	Bray–Curtis dissimilarity	*p* = 0.003, r = 0.55	*p* = 0.004, r = 0.46
	Jaccard similarity index	*p* = 0.004, r = 0.56	*p* = 0.007, r = 0.48
	Weighted UniFrac	*p* = 0.019, r = 0.37	*p* = 0.026, r = 0.31
	Unweighted UniFrac	*p* = 0.002, r = 0.51	*p* = 0.003, r = 0.4

## Data Availability

Demultiplexed fastq is available here: https://www.ncbi.nlm.nih.gov/biosample/SAMN31431713/ (accessed on 3 November 2022). The data that support the findings of this study are available from the corresponding author upon reasonable request.
